# Characterization of the Micro-Abrasive Wear in Coatings of TaC-HfC/Au for Biomedical Implants

**DOI:** 10.3390/ma10080842

**Published:** 2017-07-25

**Authors:** Pablo Guzmán, Luis Yate, Mercy Sandoval, Jose Caballero, Willian Aperador

**Affiliations:** 1 School of Engineering, Universidad Militar Nueva Granada, Carrera 11 #101-80, 49300 Bogotá, Colombia; pabloaguzman88@gmail.com (P.G.); u1801018@unimilitar.edu.co (M.S.); jose.caballero@unimilitar.edu.co (J.C.); 2 CIC biomaGUNE, Paseo Miramón 182, 20009 Donostia-San Sebastian, Spain; lyate@cicbiomagune.es

**Keywords:** micro-abrasion, wear, tantalum carbide, hafnium carbide, gold

## Abstract

The object of this work was the deposition of a Ta-Hf-C thin film with a gold interlayer on stainless steel, via the physical vapor deposition (PVD) technique, in order to evaluate the properties of different systems subjected to micro-abrasive wear phenomena generated by alumina particles in Ringer's solution. The surface characterization was performed using a scanning electron microscope (SEM) and atomic force microscope (AFM). The crystallographic phases exhibited for each coating were obtained by X-ray diffraction (XRD). As a consequence of modifying the composition of Ta-Hf there was evidence of an improvement in the micro-abrasive wear resistance and, for each system, the wear constants that confirm the enhancement of the surface were calculated. Likewise, these surfaces can be bioactive, generating an alternative to improve the biological fixation of the implants, therefore, the coatings of TaC-HfC/Au contribute in the development of the new generation of orthopedic implants.

## 1. Introduction

In the field of orthopedic implants [1], biomaterials have become a major solution due to their properties for accomplishing specific biological functions [2,3]. However, depending on the types of materials, the reaction inside the human body may vary. In different studies, orthopedic implants which have metal-metal contact surfaces show an increase in ion release generated by the influence of the aqueous environment [4,5]. As a result, the highest level of metallic ions was measured in the blood and urine [6], in comparison with implants that have a contact surface of polymer-metal, ceramic-metal, or polymer-ceramic. Nevertheless, the systems with a contact surface of metal-metal generate a lower volume of periprosthetic inflammation in the tissue due to the lower liberation of particles that are produced with the wear with respect to other systems mentioned previously [7,8]. Therefore, the interest for studying the mechanical wear has increased since it became one of the main factors that cause implant failure or, in other cases, that can contaminate the environment that contains it in the long-term [9].

As a result of the requirement for improving the wear resistance, the PVD technique has allowed the deposition of thin films that modify the physical and chemical properties of the surface without modifying the mechanical properties of the substrate [10,11,12]. The use of tantalum as a biomaterial has good acceptance due to improvement in the corrosion resistance and wear resistance, and it also has good biocompatibility inside the human body [13,14]. Likewise, different studies have shown the significant applicability of tantalum as a coating because it exhibits similar mechanical properties as bone, such as high hardness and flexibility. Moreover, tantalum allows the regeneration of the biological material when it coats the implant [15,16].

Another material that is implemented in the biomaterials field is hafnium, as the addition of this material stimulates an improvement in wear resistance and surface integrity against deformation [17,18]. Furthermore, this material enhances the tensile strength and modulus of elasticity, decreasing the coefficient of friction [4,19]. Finally, as with tantalum, hafnium increases the corrosion resistance and has a good biocompatibility response [20].

The use of solid lubricants as a thin film has increased since traditional lubricants cannot reach some restrictions in the environments that require specific conditions, such as extreme temperatures [21], vacuum environments, or a high grade of purity as required inside the human body [22]. The solid lubricants are generated from “soft metals”, such as gold and silver [15], and through the PVD technique on different substrates [23,24]. Likewise, this type of lubricant is applied on various precision mechanisms, because its proper function depends on its pieces’ surface conditions. However, the characteristics of the selected lubricant may vary depending on the application and the conditions it will have to contend with when it is in service [25].

In the other hand, in thin films there exists a correlation between surface hardness and the coefficient of friction. At a higher hardness the coefficient of wear is lower, improving the response to wear phenomena [26,27]. To improve the surface hardness and reduce the presence and propagation of cracks, the process of carburization through the PVD technique has been highly applied as the addition of carbon results in a harder, more stable, and resistant film [28,29,30].

The aim of this work was to perform a deposition on 316LVM stainless steel with a coating of HfC/Au, TaC/Au, and mixtures of TaC-HfC in which the behavior of micro-abrasion wear in simulated biological conditions was evaluated. It was determined that the coatings improve against wear.

## 2. Materials and Methods

For the deposition of different coatings targets 50.8 mm diameter comprised of gold, tantalum, and hafnium with 99.95% purity and a carbon target with 99.999% purity were used in a confocal configuration at 0.4 Pa pressure in argon. For the deposition of different films the non-reactive magnetron sputtering technique in an AJA-ATC 1800 UHV equipment with a base pressure of 10^−7^ Pa was used.

Previously, the deposition of the films on substrates of 316LVM stainless steel were cleaned with ethanol and, subsequently, with plasma at a radio frequency (RF) with a power of 25 W in a pure argon environment at a pressure of 4 Pa for 10 minutes. Prior the deposition of the gold film, and to enhance the adhesion of the film to the substrate, was deposited a thin metallic layer of Ta-Hf with a thickness of approximately 20 nm between the substrate and the gold film by applying an RF power of 100 W to each Ta-Hf target. The gold films were obtained by applying a direct current (DC) power of 150 W to the gold target with a substrate rotation of 80 rpm for 20 minutes at room temperature, obtaining a thickness of 0.8 µm.

For the deposition of the different Ta-Hf-C films , the power applied to the targets was varied in the following way: 100-0 W (TaC), 70-30 W (70TaC 30HfC), 30-70 W (30TaC 70HfC), and 0-100 W (HfC), while the power of the carbon target remained at 380 W. Deposition of the different films was carried out with a target-substrate distance of 15 cm and at a temperature of 300 °C, with a negative RF voltage of 50 V.

The characterization of the crystalline phases was carried out on an X-ray diffractometer in a Bragg-Brentano configuration with a classic scintillation detector. The X-ray tube was made of copper with a Kα1 line of 1.540598 Å and Kα2 line of 1.544426 Å, with an incident ray radius of 240 mm. The voltage difference applied was 40 kV with an intensity of 30 mA, and the sweep interval was from 20 to 90°. The counting time designated for this test was stipulated at 2 s with a step size of 0.0400, for a total of 2125 points. Phase identification was performed in HighScore Plus software (Version 4.6a, Panalytical, Almelo, The Netherlands) with the ICSSD [31] and COD databases.

**Figure 1:**
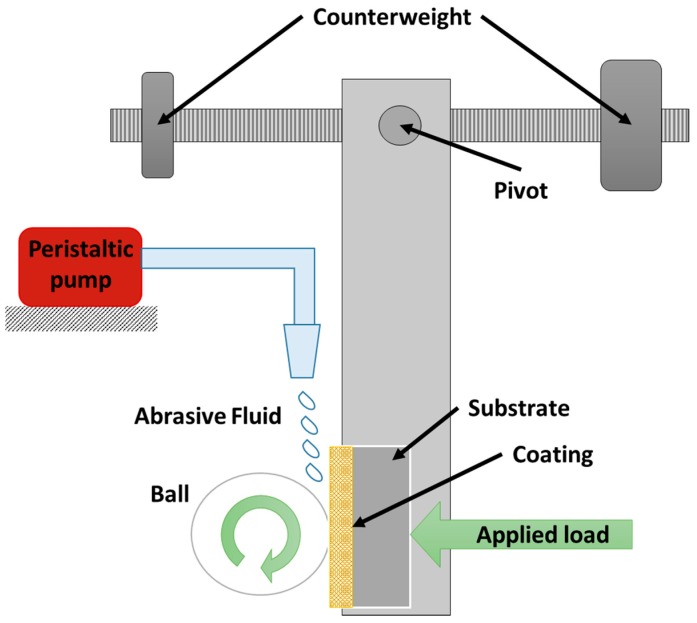
Schematic of the micro-abrasion test apparatus which allow applying a normal load between the coated samples and the ball also shows how the alumina particles are added to the system.

The micro-abrasion test was carried out in equipment that allows the simulation of the wear between two contact surfaces. The apparatus has a lever arm in which the sample is held and a fixed sphere between two coaxial axes. Through the movement of the counterweight, it is possible to regulate the load applied to the surface and the sphere, which is rotated by a DC motor with an encoder that guarantees control over the speed and number of cycles performed. Furthermore, a peristaltic pump adds a third body in the form of abrasive particles suspended in a solution to generate the micro-abrasive processes, as shown in Figure 1.

With the aim to simulate biological conditions of a joint replacement by means of an ultra-high molecular weight polyethylene sphere and Ringer’s solution with alumina particles in suspension, the test parameters are shown in Table 1.

For the estimation of the wear rate parameter, a model that relates the dimensions of the wear path performed on the surface of the sample with a wear constant was considered. According to the authors Gee et al. [32,33], the wear volume V is defined by:

(1) $$V=\frac{\pi b^{4}}{64R}\;para\;b\ll R$$

where *b* is the average diameter of the scar and *R* is the radius of the sphere with which the test is performed. Relating Equation (1) with Archard's law of wear, Equation (2) is obtained, where *K* is the wear constant, *S* is the sliding distance, and *N* is the normal load applied:

(2) $$K=\frac{V}{SN}$$

The surface was observed by a JEOL NeoScope JMC-5000 scanning electron microscope operating under high vacuum at 10 kV. Superficial roughness was evaluated by measuring the arithmetic average of the roughness profile (Ra) through a NaioSurf atomic force microscope reference, and the measurement was carried out with an image size of 46.2 μm × 46.2 μm with a 1 s time/line, a resolution of 256 points/line, and a setpoint of 20 nN using the contact mode with a silicon tip coated with aluminum. The measurements were performed in two different zones: The first zone of analysis was obtained prior to each wear test to characterize the surface. In the second zone, the analysis was carried out in the central area generated by the sphere to identify the wear suffered by the coatings. All of the measurements were repeated three times. Using SPIP Image Metrology software, the images were analyzed to obtain the average roughness for each system before and after the micro-abrasion test [34].

## 3. Results and Discussion

### 3.1. XRD Characterization

**Figure 2:**
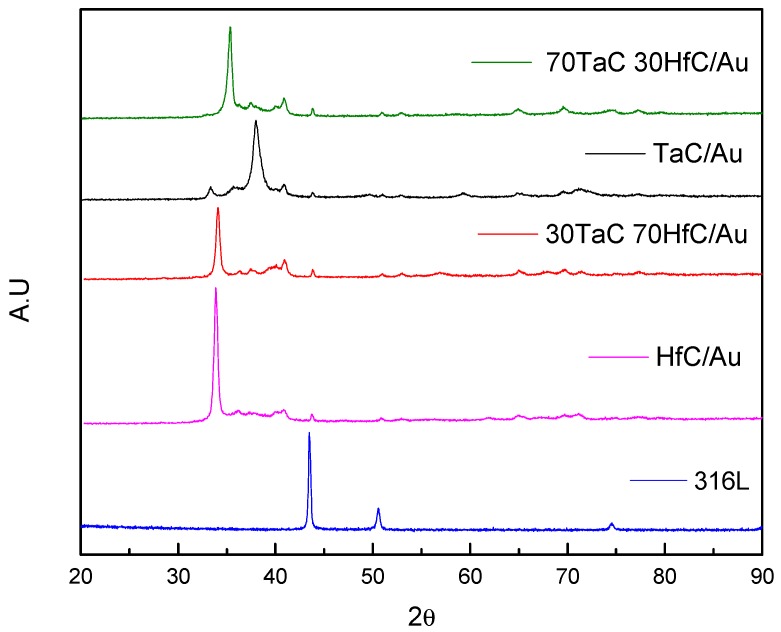
Diffraction pattern of the substrate and the different samples of TaC/Au, HfC/Au, 30TaC/Au, and 70TaC-30HfC/Au studied using XRD.

In the interpretation of the crystalline phases of the coatings, the substrate was measured without any coating to identify the characteristics’ peaks that it presented, and subsequently identified the characterization of the different samples. Figure 2 shows the diffraction patterns obtained for each sample in which 316LVM stainless steel exhibited reflections at 43.7°, 50.52°, and 74.47°, attributed to the iron and chromium elements of the substrate and are also replicated in the measurement of the crystalline phases for most of the samples,. In films with a high content of hafnium, such as HfC/Au and 30TaC-70HfC/Au, the identification of reflections at 36.19°, 39.4°, and 69.37° were related with the Hf_2_Au_1_ phase with planes in (103), (110), and (213), with a tetragonal crystalline system. On the other hand, the samples with a high content of tantalum, such as TaC/Au and 70Tac-30HfC/Au, show a match at 35.01° and 40.75°, which are attributed to the phase of Ta_4_C_3.04_, with (111) and (020) planes in a cubic crystalline system.

**Figure 3:**
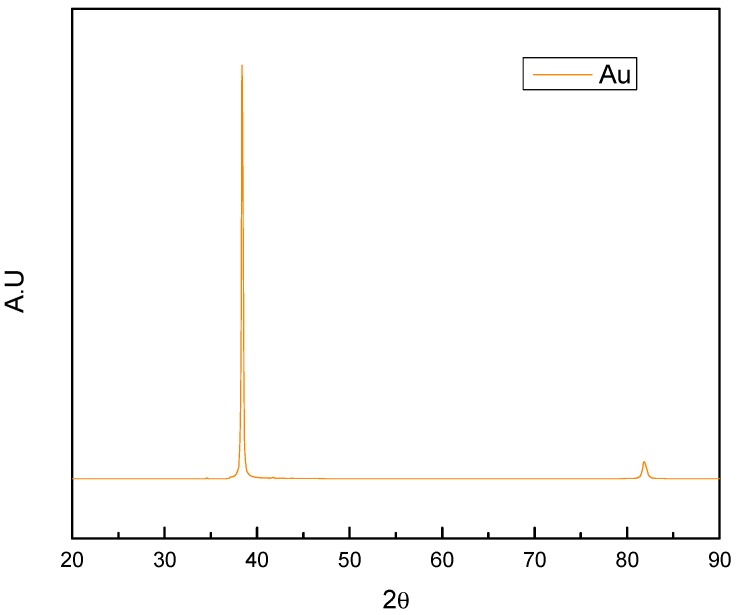
Gold coating pattern studied using XRD.

In the samples with gold coatings, Figure 3 identified reflections at 38.068° and 82.05° with planes in (111) and (222) in a cubic crystalline system. Likewise, this sample showed a high crystallinity because the count obtained for this sample was much higher in comparison to the other samples. Moreover, gold phases were identified in the samples of TaC/Au and HfC/Au at an angle of 38.068°, which is more evident for the sample of TaC/Au.

### 3.2. Wear Estimation

**Figure 4:**
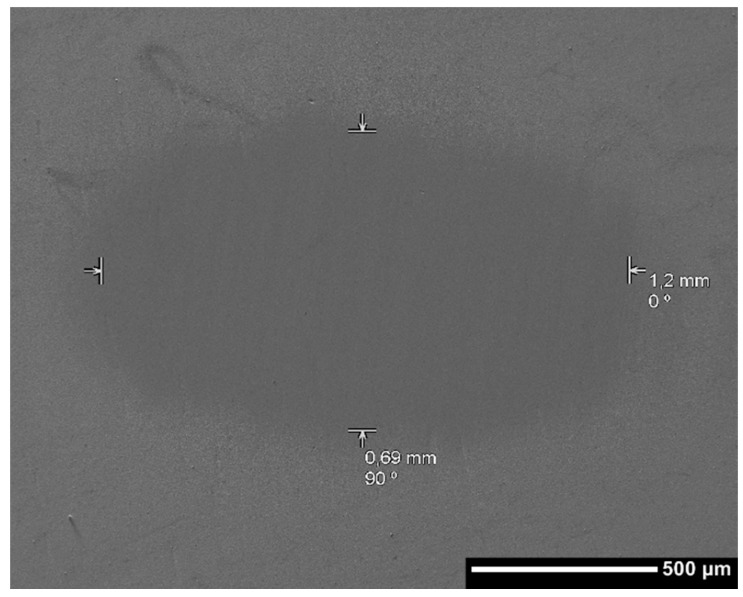
Micrograph of the wear scar length measure through SEM technique.

Through the scanning electron microscope technique the length of the scar generated by the wear process was measured. The measurement was carried out parallel and perpendicular to the direction of rotation of the sphere, as shown in Figure 4. These lengths were averaged to obtain an estimated diameter of the scar for each sample, as shown in Table 2.

**Figure 5:**
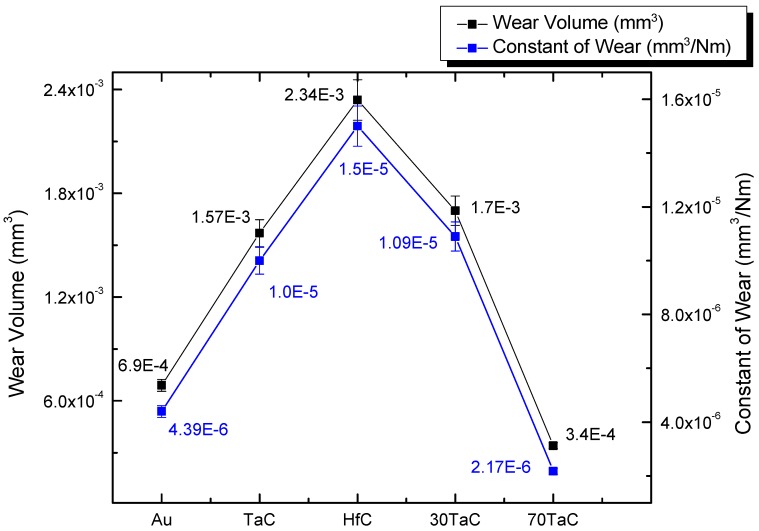
Contrast between the wear volume and constant of wear for each coated system.

Taking into account the average lengths, all of the tests were carried out with a normal force of 3 N and a sliding distance of 78.53 m, as used in Equations (1) and (2), to obtain the wear volume and the wear constants as shown in Figure 5.

The coating of 70Tac 30HfC/Au shows the lowest constant wear value, followed by the gold sample, indicating a superior behavior against the micro-abrasion wear compared with the other systems. On the other hand, the coatings’ performances of TaC, HfC, and 30TaC 70HfC were similar, where the highest value was exhibited by the HfC sample. Nevertheless, all of the coatings showed values in their constants lower than 10^−4^, which indicated great performance since the wear was moderate [35].

### 3.3. Morphological Analysis

After the test was carried out measurements on the wear scar through SEM and AFM techniques were conducted to morphologically characterize the surface.

#### 3.3.1. SEM

**Figure 6:**
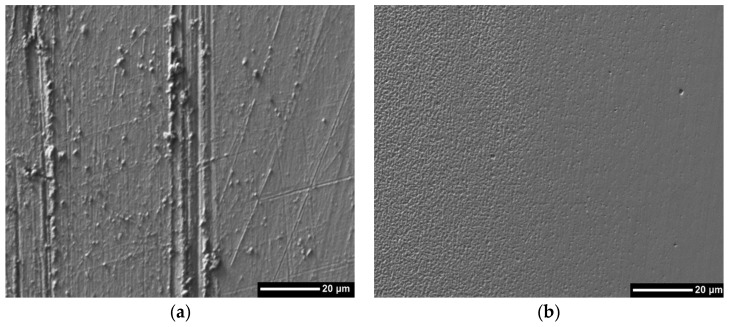
Micrograph of the most affected area by micro-abrasion tests studied by SEM technique. (**a**) Au, and (**b**) TaC/Au.

In Figure 6, the micrographs of the evaluated surface were observed with a magnification of 1000× and only the two most relevant micrographs are shown since a significant change is not evident in comparison with the other systems. From the micrographs it can be seen that the coatings protect the substrate from wear phenomena, which can be identified by the total removal of the coating until access to the substrate is achieved.

In Figure 6b good performance against wear is observed, since the surface damage was minimal. Nevertheless, the coating of gold in Figure 6a shows the start of the mechanism of wear by micro-abrasion, and the micrograph reveals small grooves defined in the direction of the movement of the alumina particles, which is characteristic of the grooved wear regime.

#### 3.3.2. AFM

**Figure 7:**
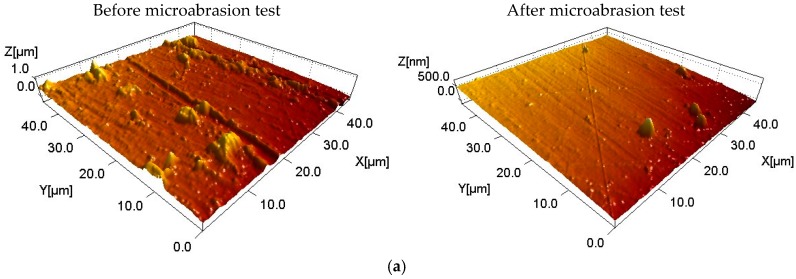

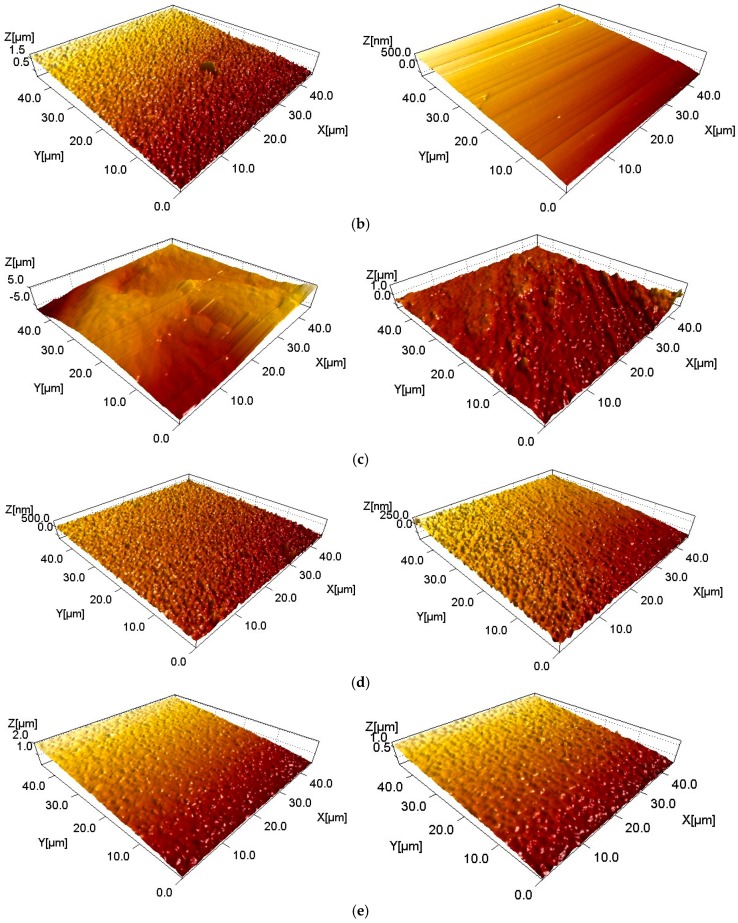
Surface characterization of each sample using AFM technique before and after micro-abrasion test (**a**) Au, (**b**) TaC/Au, (**c**) HfC/Au, (**d**) 30TaC-70HfC/Au, and (**e**) 70TaC-30 HfC/Au.

**Figure 8:**
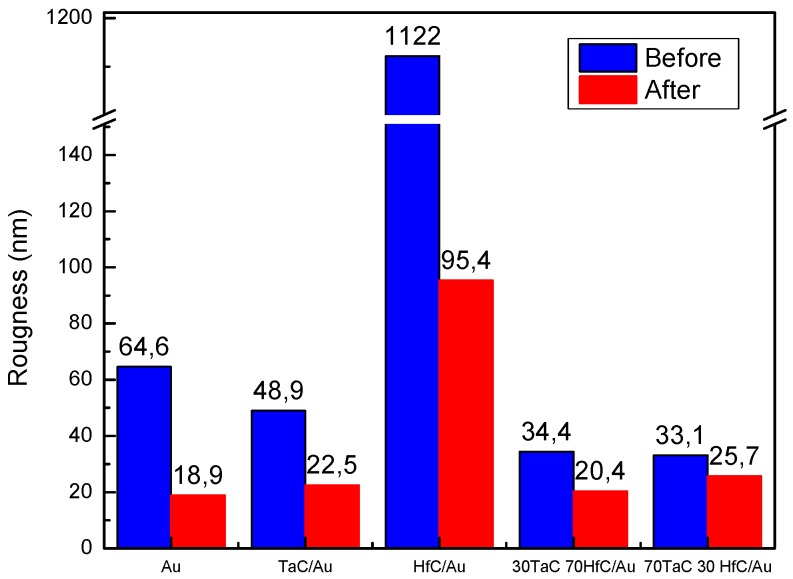
Quantitative variation of the roughness measurements before and after micro-abrasion test using AFM technique and analyzed by SPIP Image Metrology software.

In the micrographs obtained by AFM, Figure 7 shows the surface characterization of the different samples. All of the surfaces exhibited a variation in the roughness, as shown in Figure 8, that was induced by the effect of the sphere with the addition of the alumina particles. After the micro-abrasion test three types of surface variation was observed, with the largest change of the roughness value shown by the sample of HfC/Au (Figure 8), which was related to the morphological change associated with the ridge degradation, interpreted for this case of study as severe variation.

On the other hand, samples of TaC/Au and gold showed a superficial variation considered average as a consequence of a morphological change that eliminated material and homogenized the surfaces. Finally, samples with a combination of TaC-HfC exhibited a slight morphologic change related with the other systems in addition to showing a lower roughness value.

## 4. Conclusions

In the characterization of the crystalline phases it can be concluded that the gold coating had a major crystalline orientation compared with the other coatings since the gold thin film obtained by PVD showed a crystalline cubic structure, reflected in the counts obtained for this sample.

Regardless of the varying deposition parameters, the crystalline phase of Hf_2_Au_1_ tends to replicate in both the pure HfC/Au samples, as well the 30TaC-70HfC/Au samples, and also the Ta_4_C_3.04_ phase was identified for the coatings of TaC/Au and 70TaC-30HfC/Au.

All of the systems evaluated in this study had a good behavior against the phenomenon of micro-abrasion wear in the presence of Ringer’s solution, which is evidenced by the low values of the wear constants. Furthermore, although the HfC coating presented the highest value in terms of the wear constant, it shows advantages when it is in service since its roughness decreases significantly, generating a lower friction.

Although the Au film showed signs of grooved debris by micro-abrasive processes, it was also one of the systems with the best behavior, due to exhibiting the second-lowest wear constant value. On the other hand, after the system of HfC/Au, the gold system showed a greater decrease in its roughness and had the lowest value related to the others coatings.

Moreover, the coatings obtained via PVD allows generating a thin film that functions as a protective barrier against wear phenomena which is one of the main causes of failure of an implant due to deterioration and development of surface wear and cracks propagation. This degradation can extend through all the surface of the material and is important to avoid this kind of circumstance because in extreme cases can affect the structural integrity of the implant leading to the need of remove the implant.

## Figures and Tables

**Table 1:** Test parameters.

Specifications of the Micro-Abrasion Test	
Material of sample	316LVM stainless steel, coatings of TaC/Au, HfC/Au, Au, 70TaC 30HfC/Au, and 30TaC 70HfC/Au
Material of sphere	Ultra-high molecular weight polyethylene sphere UHMWPE (25 mm diameter)
Size of the particles	Alumina, 0.3 µm
Solution	Ringer's balanced salt solution
Concentration of particles	0.02% (10 g alumina in 500 ml of Ringer’s solution)
Velocity	40 rpm
Normal load	3 N
Number of cycles	500 revolutions
Sliding distance	78.53 m
Flow rate	30 mL/min

**Table 2:** Measurement of length.

Sample	Perpendicular Length (mm)	Parallel Length (mm)	Average Length (mm)
Au	0.59	0.95	0.77
TaC	0.69	1.2	0.945
HfC	1.1	0.99	1.045
30TaC 70 HfC	1.2	0.73	0.965
70TaC 30HfC	0.99	0.3	0.645
